# Primary Varicella-Zoster Virus Infection Complicating the Third Trimester of Pregnancy

**DOI:** 10.7759/cureus.103060

**Published:** 2026-02-05

**Authors:** Marcos Sosa, Abigail I Bagdasaryants, Alwyn J Mathew, Jacqueline Q Sosa

**Affiliations:** 1 Department of Obstetrics and Gynecology, Houston Methodist Hospital, Houston, USA; 2 Department of Pediatrics, Dell Children's Medical Center, Austin, USA

**Keywords:** acyclovir, chickenpox, neonatal, pregnancy, rash, vaccination, valacyclovir, varicella-zoster

## Abstract

Varicella-zoster virus, colloquially known as chickenpox in the United States, complicating pregnancy has become a rare diagnosis following the introduction of the varicella-zoster vaccine. Despite a lower incidence of chickenpox than in the past, those providing prenatal care should keep a high index of suspicion for varicella-zoster among patients presenting with a vesicular rash and no history of having received the varicella-zoster vaccine. This report reviews the case of a 28-year-old gravida three at 35 weeks gestation who presented with concern for a full-body vesicular rash and pruritus. The evaluation, diagnosis, and medical management of varicella-zoster infection in pregnancy are discussed.

## Introduction

Varicella-zoster virus (VZV) is a double-stranded DNA virus from the Herpesvirus family, and is the etiologic cause of varicella, commonly known as “chickenpox,” via primary infection [1]. Varicella is an extremely contagious, often childhood exanthem, characterized by pruritic, vesicular maculopapular lesions preceded by prodromal, flu-like symptoms of malaise, fever, and headache [1]. It is transmitted through droplets or direct contact with skin lesions [1]. Immunity is acquired through natural infection or vaccination. Disease is typically self-limited, and complications rarely manifest outside of immunocompromised individuals. However, pregnancy complications can occur.

Varicella infection in pregnancy can cause harmful effects to both the mother and the fetus. The risk of developing VZV pneumonia in pregnancy has been cited as high as 10-20%, which may be most severe during the third trimester [2]. Fetal sequelae occur by trans-placental transmission and depend on the trimester in which infection occurs. Infection within the first 20 weeks of gestation can cause congenital varicella syndrome, characterized by a constellation of signs and symptoms including limb hypoplasia, neurological abnormalities, eye disease, and cicatricial skin lesions [2,3].

Maternal varicella infection in the last four weeks of pregnancy has a high risk of perinatal transmission of infection to the newborn [2]. Specifically, maternal infection between five days before delivery through two days after delivery can lead to devastating consequences to the newborn who has not yet acquired maternal antibodies, with fulminant infection causing mortality in up to 20% of newborns not treated with acyclovir [3]. Infants born to mothers who develop varicella in this time frame should receive varicella-zoster immune globulin (VZIG) [4]. Some professional societies, including the Royal College of Obstetricians and Gynecologists, recommend delaying scheduled delivery for seven days after the onset of maternal varicella rash [2].

In this report, we discuss one case of a pregnant patient with no prior history of varicella-zoster infection or vaccination who developed vesicular lesions on her abdomen for whom scheduled cesarean delivery had to be delayed.

## Case presentation

A 28-year-old gravida three at 35 weeks of gestation presented to the obstetrical care provider with a concern for a new onset full-body rash. Her prior pregnancy was complicated by intrahepatic cholestasis of pregnancy (ICP), for which she was induced at 37 weeks of gestation. She was also diagnosed with ICP in the current pregnancy at 30 weeks of gestation. Bile acids were elevated at 199 µmol/L (normal <10 μmol/L) at 30 weeks of gestation. She had an associated transaminitis with an aspartate aminotransferase (AST) of 80 IU/L (normal range 8-33 IU/L) and an alanine aminotransferase (ALT) level of 204 IU/L (normal range 7-56 IU/L). She was followed by Maternal Fetal Medicine (MFM) for weekly antenatal testing for the diagnoses of ICP and insulin-dependent diabetes. MFM recommended delivery at 37 weeks of gestation.

**Figure 1:**
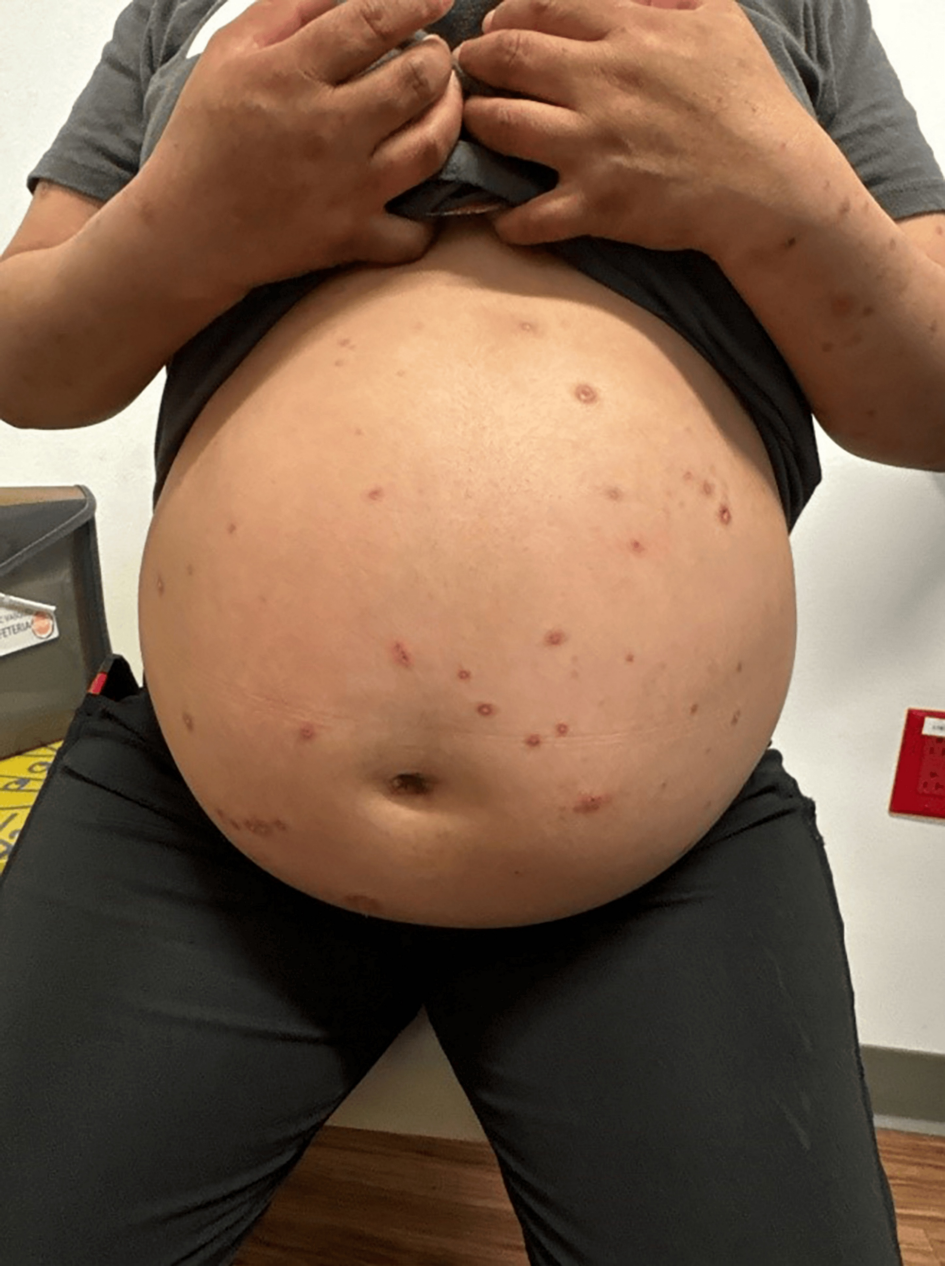
Varicella (chickenpox) on the abdomen of a patient at 35 weeks gestation.

**Figure 2:**
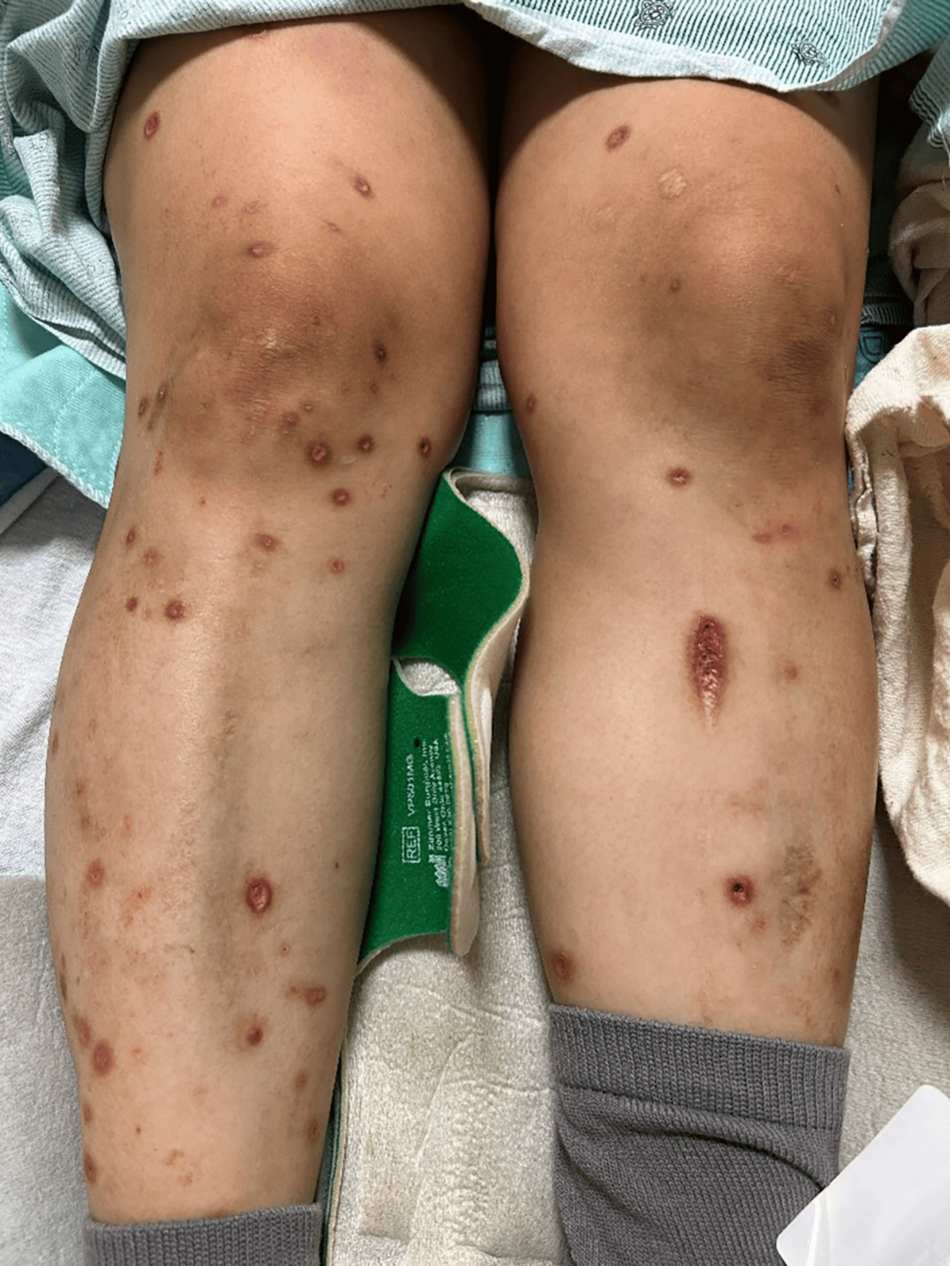
Varicella (chickenpox) lesions affecting the lower extremities.

The patient had been experiencing pruritus beginning at 30 weeks of gestation. At 35 weeks of gestation, she noticed multiple, extremely pruritic vesicles developing on her abdomen and extremities. Figure 1 is an image of the vesicular rash on the patient's gravid abdomen. Dermatologic evaluation revealed vesicular lesions ranging from 2mm to 11mm in size on the abdomen, back, vulva, and bilateral lower extremities. The lesions were erythematous with evidence of excoriations. A lesion on the left lower leg was open, erythematous, and pustulent, concerning for an overlying skin infection. Figure 2 is an image of the vesicular lesions of the lower extremities. A viral culture was taken from a lesion on the abdomen and sent to the laboratory for evaluation. 

Detailed medical history revealed that the patient had never received a varicella-zoster vaccine. The patient had a varicella antibody titer level drawn with her routine prenatal blood work in the second trimester, and she was found to be non-immune. Given a high suspicion for varicella infection, the patient was started on valacyclovir while the culture results were pending. Clindamycin was prescribed for the left leg cellulitis. The culture ultimately resulted as positive for varicella-zoster. Varicella-zoster IgM antibodies were positive in the bloodwork obtained. Varicella-zoster IgG antibodies were negative.

The patient had been previously scheduled for a repeat cesarean delivery at 37 weeks of gestation secondary to ICP. However, MFM recommended that delivery take place after the lesions began to crust over. This occurred at 37 weeks and four days of gestation. An ultrasound performed by MFM the day before delivery showed an estimated fetal weight in the 30th percentile, a biophysical profile score of eight out of eight, and no fetal anomalies. A repeat cesarean delivery was then performed. A male infant with Apgar scores of 8 and 9 at one and five minutes, respectively, was delivered without complications. The neonatology team was consulted for management of the neonate. Neonatology staff was present for evaluation of the neonate at delivery and noted no evidence of varicella-zoster infection. Their recommendations highlighted that there was no risk associated with skin-to-skin contact of the neonate with the mother, and that no isolation precautions needed to be taken between the mother and neonate or between the neonate and other infants on the unit. They also highlighted that the patient presented outside the window to receive immunoglobulin.

## Discussion

Fortunately, symptomatic VZV infection has become rarer among adults since the introduction of the varicella-zoster vaccine. The United States was the first country to adopt the live, attenuated varicella vaccine for the prevention of varicella following its 1995 licensure (VARIVAX, Merck & Co., Inc.) [5]. A single dose is 82% effective at preventing varicella infection and almost 100% effective at preventing severe varicella infection [6].

Internationally, multiple studies have looked at rates of immunity to varicella among pregnant women, but no large-scale studies have been performed to look at immunity rates in the United States. For example, a study in Norway showed 98.6% of pregnant women were seropositive for varicella-zoster IgG antibodies among a sample of 1,184 pregnant women, and in Alberta, Canada, 95.8% of pregnancy screenings showed seropositivity out of a sample size of 454,592 [7,8].

Primary varicella infection in a pregnant woman most often results in the birth of a healthy newborn. However, contracting varicella during the first two trimesters may lead to fetal death and/or congenital varicella syndrome (CVS). CVS can result in a range of teratogenic effects including eye, gastrointestinal, and urogenital abnormalities. Limb hypoplasia, cutaneous scarring, and central nervous system damage are other sequelae. The highest risk for spontaneous abortion or CVS occurs within the first 20 weeks of gestation but is estimated to be less than 2% [9]. There are some case reports of CVS developing between 21-28 weeks of gestation, but no risk of CVS has been identified in the third trimester [10]. 

Management of primary VZV infection in pregnancy depends on the gestational age of the pregnancy. For all gestational ages, management includes symptomatic control. Oral acyclovir (800 mg) five times a day for seven days should be offered if the patient presents within 24 hours of the rash’s onset, particularly in patients between 20-40 weeks of gestation [2]. Evidence of severe infection fulminating in varicella pneumonia will require inpatient intravenous anti-viral treatment. MFM referral for serial fetal monitoring at 16-20 weeks and/or five weeks after onset of the rash should be performed to evaluate for viral effects in the developing fetus [2]. For maternal VZV infections from 36 weeks of gestation to seven days after delivery, elective delivery should be delayed for seven days from onset of rash if other obstetric indications permit [2]. In the case presented above, the timing of delivery occurred after a multi-disciplinary discussion between maternal-fetal medicine, labor and delivery, and neonatal intensive care unit providers. 

In the third trimester, maternal varicella infection can lead to severe neonatal disease if the maternal rash first develops five days before delivery and up to two days after delivery [11]. The severity is due to the lack of maternal varicella-specific IgG antibody transfer to the infant and the immaturity of the neonatal immune system [10]. Infection may occur by ascending infection from the birth canal, transplacental viremia, respiratory droplet exposure, or through direct contact with the non-crusted skin lesions during or after delivery [12]. These infants should ideally receive VZIG or intravenous immunoglobulin (IVIG) immediately after birth, or as soon as maternal symptoms appear within the two days following delivery. VZIG, which provides varicella-specific IgG, is the preferred treatment. Typically, infants born during this period appear well at birth but may develop symptoms shortly after. They are at risk for manifestations including the classic varicella skin lesions, pneumonia, hepatitis, encephalitis, liver failure, and thrombocytopenia. Intravenous acyclovir should be initiated if the infant exhibits signs of illness.

Infants born more than seven days after the initial appearance of the maternal rash are not at high risk of severe disease. These infants may have lesions at birth or develop them within the first several days of life. They are protected from severe varicella disease due to maternal VZV antibodies that have had sufficient time to cross the placenta [10]. These infants should be treated with acyclovir if they manifest any active varicella infection [9].

## Conclusions

VZV infection in pregnancy has become a rarity since the adoption of routine varicella-zoster vaccination. However, the patient presentation discussed in this article is evidence that obstetrical providers must be educated on the signs and symptoms of VSV, especially among those who have not received the VSV vaccine or who do not have immunity as evidenced by laboratory findings. Preventive vaccination before a woman becomes pregnant remains the best defense against the potentially devastating fetal effects of varicella. However, the true rate of immunity in pregnant women in the United States is not well documented. Providers who have never seen a chickenpox infection due to high vaccination rates in their communities should continue to remain vigilant and be able to recognize the dermal lesions and associated symptoms. Management of pregnant patients with VZV infection requires a multidisciplinary approach involving care by obstetricians, maternal-fetal-medicine specialists, and neonatologists. Management of the pregnant patient depends on gestational age as well as the length of time the patient has had the active infection. Oral anti-viral medication, including acyclovir and valacyclovir, should be considered in patients with recent-onset chickenpox infection. Management of a newborn depends on timing of maternal infection, timing of delivery, and neonatal signs of active infection. Data should be kept on the geographic area of a chickenpox outbreak. Vaccination before or after pregnancy should be recommended.
